# Reflections on the dynamic zero-COVID policy in China

**DOI:** 10.1016/j.pmedr.2023.102466

**Published:** 2023-11-22

**Authors:** Zaihua Ba, Yuqi Li, Jiao Ma, Yining Qin, Jinzhu Tian, Yixiang Meng, Jiarong Yi, Yingze Zhang, Fei Chen

**Affiliations:** Jining Medical University, 133 Hehua Rd, Jining 272067, China

**Keywords:** China, COVID-19, Dynamic zero-COVID policy, Lockdown, Omicron, SARS-CoV-2

## Abstract

The coronavirus disease 2019 (COVID-19) pandemic has posed a serious threat to global healthcare and economy. In order to curb its spread, China adopted the dynamic zero-COVID policy, aiming to diagnose and isolate cases and close contacts as soon as possible. However, there is a controversy about the impact of isolation measures on social order, including the economy, personal employment and public mental health. Therefore, this review discusses and analyzes in detail the advantages and challenges of implementing dynamic zero-COVID policy. Although this public health policy might cause a shock to the economy in the short term, China still achieved a continued healthy economic performance with stable unemployment and strong export growth. Moreover, the rates of infection and mortality in China were lower than those in the United States and the European Union. However, due to the high transmissibility and low pathogenicity of the Omicron variant and prolonged lockdown-induced psychological damage, people questioned the effectiveness and necessity of this policy. Now that China has adjusted its policy in a timely manner, but many problems still remain unsolved. Some practical suggestions in terms of mental health, vaccine development, drugs supply, and economic recovery are put forward at the end of our paper to minimize negative impacts and provide a reference for future efforts.

## 1 Introduction

The coronavirus disease 2019 (COVID-19) continues to be prevalent worldwide. Various strategies have been taken to prevent and control it in different countries, which can be broadly divided into living with COVID and dynamic zero-COVID policy. Specifically, the dynamic zero-COVID policy sets zero deaths as its goal and achieves by containing transmission through short-term lockdown, followed by the strict find, test, trace and isolate methods, while living with COVID is framed as “flattening the curve” by establishing targets for community transmission and imposing restrictions to reduce spread until those targets are met (Baker et al., 2020); (Jecker and Au, 2022). With the appearance of available antivirals and vaccines, some countries such as Singapore and New Zealand, have gradually transitioned from the dynamic zero-COVID policy to living with COVID. Due to the differences in sociocultural, political and economic contexts between countries, there is currently no consensus on the best public health policy for COVID-19.

The period from January to April 2020 was the emergency containment phase of the COVID-19 epidemic in China (Chen et al., 2021). Wuhan, the epicenter of the epidemic, implemented unprecedented prevention and control measures including closing the city and restricting the movement of people (Yao et al., 2021). Strict home quarantine, education on personal preventive behaviors, delaying the start of enterprises and other measures were also taken in other places to prevent further expansion of the epidemic (Horton, 2020); (Wilder-Smith and Freedman, 2020). After this phase, China entered a normalization stage of epidemic prevention and control (Liang et al., 2021). Local governments established and improved the transformation mechanism of emergency response and strengthened monitoring and early warning, which effectively controlled sporadic cases or aggregated outbreaks (Lin, 2022). However, the highly transmissible Delta variant hit the country in August 2021 (García-Herrero, 2022). To reduce the possibility of another outbreak of the epidemic, China doubled down on containment policies by formally introducing the concept of dynamic zero-COVID policy. Notably, this policy has to a certain extent disrupted the normal order of society and has been questioned in the past year. Exposed contacts isolated at home had to purchase food and necessities through a new program called group-buying (Cheng, 2022). However, sometimes it was difficult to ensure timely delivery of supplies (Burki, 2022). In some places, people’s daily routine was seriously affected by simple and violent measures such as road blocking, road digging, frequent large-scale mandatory PCR testing and so on (Wang and Huang, 2022). After a long period of lockdown, many people felt physically and emotionally drained and wanted urgent changes to this policy (Burki, 2022).

On December 7, 2022, Comprehensive Group of Joint Prevention and Control Mechanism in response to COVID-19 epidemic situation of the State Council of China announced 10 new prevention and control measures, making major adjustments to its COVID-19 response (Burki, 2022). Under this policy, individuals with mild or asymptomatic disease can be isolated at home rather than in designated quarantine facilities. Access to public places is no longer restricted by digital health pass. Moreover, lockdown will be highly focused in specific buildings rather than whole municipal areas or cities, and will be relaxed after 5 days without any new cases. Subsequently, the National Health Commission renamed the Chinese term for the disease from “novel coronavirus pneumonia” to “novel coronavirus infection” and downgraded the management to Class B (Luo et al., 2023). Starting from January 8, 2023, China won’t take any quarantinable communicable disease control measures for inbound personnel and goods, and will gradually resume tourism in an orderly manner (Newswire, 2022). Although China’s dynamic zero-COVID policy has ended, the handling of similar public health emergencies now and in the future can expect the support of experiences and lessons we have learned in the past three years. Therefore, there is still a need to seriously summarize and rationally reflect on the effects of past epidemic prevention policy and its implementation measures, and provide useful suggestions for the government and individuals in the new situations, so as to improve the policy system for epidemic control and public’s satisfaction and confidence.

## 2 Advantages of the dynamic zero-COVID policy

**Fig. 1:**
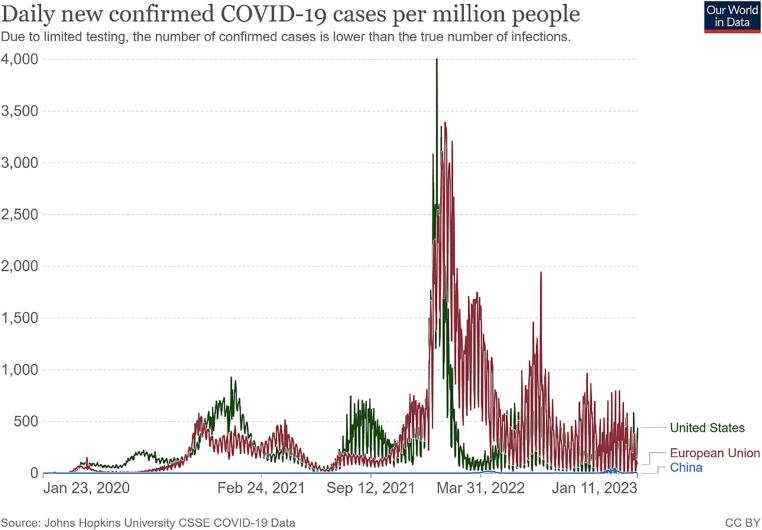
Daily new confirmed COVID-19 cases per million people in the United States, European Union and China **Source:** Johns Hopkins University CSSE COVID-19 Data.

**Fig. 2:**
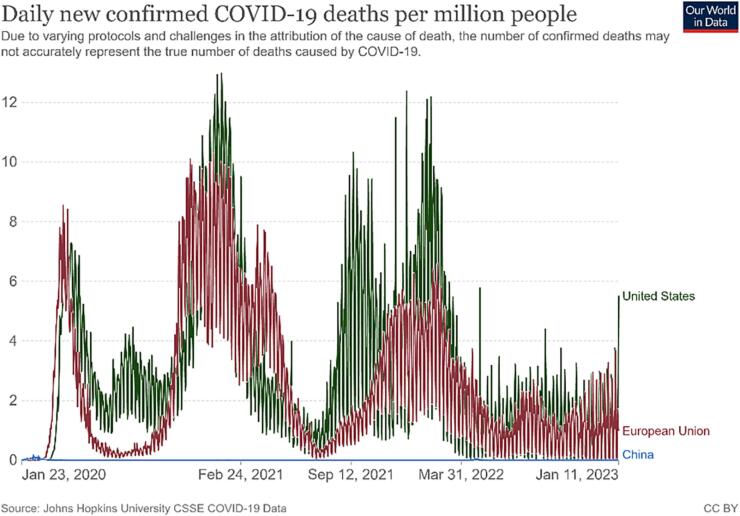
Daily new confirmed COVID-19 deaths per million people in the United States, European Union and China **Source:** Johns Hopkins University CSSE COVID-19 Data.

**Fig. 3:**
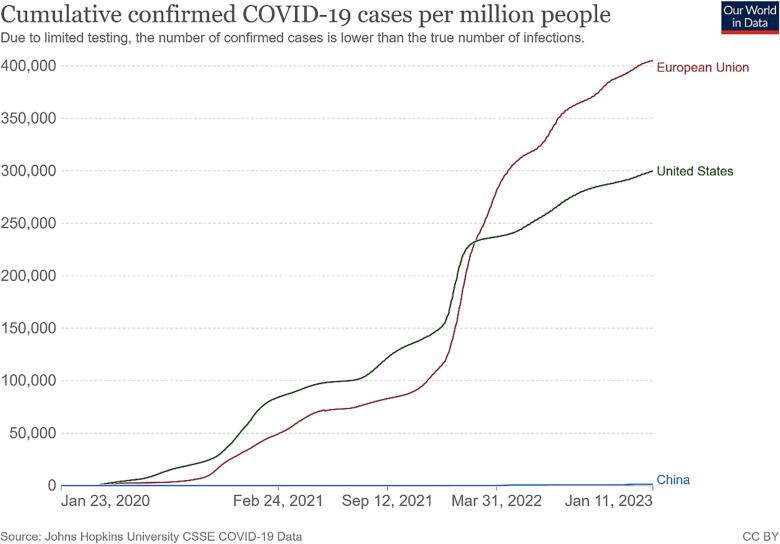
Cumulative confirmed COVID-19 cases per million people in United States, European Union and China **Source:** Johns Hopkins University CSSE COVID-19 Data.

**Fig. 4:**
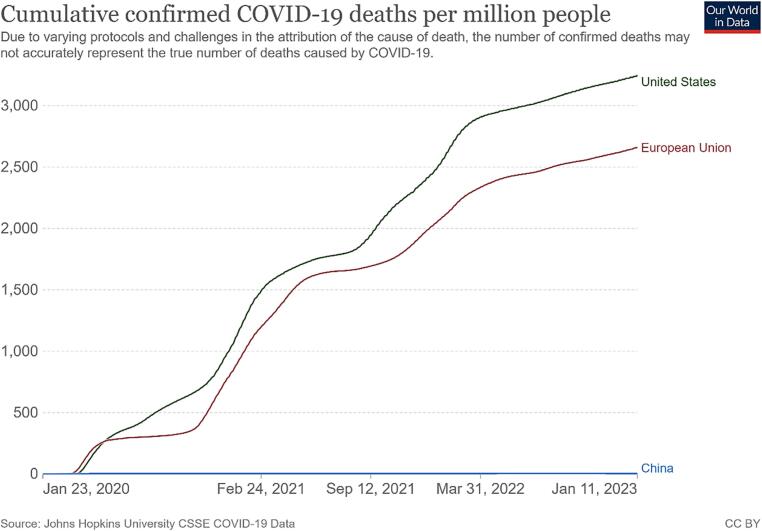
Cumulative confirmed COVID-19 deaths per million people in United States, European Union and China **Source:** Johns Hopkins University CSSE COVID-19 Data.

**Fig. 5:**
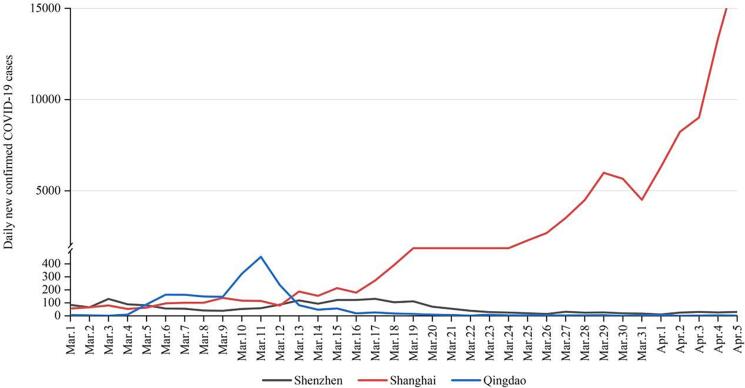
Daily new confirmed COVID-19 cases in Shanghai, Qingdao and Shenzhen from 1 March to 5 April 2022 **Source:** National Health Commission of the People’s Republic of China.

There is no doubt that relaxing isolation and distancing requirements would create more opportunities for the virus to spread (Feng et al., 2022). Many countries attempting to co-exist with the circulating virus have experienced several large-scale outbreaks of the COVID-19 epidemic (Cavalcante da Silva et al., 2021); (Hassan and Mahmoud, 2021); (Salyer et al., 2021). Fig. 1, Fig. 2 showed daily new confirmed COVID-19 cases and deaths per million people of the countries and regions implementing different policies from 23 Jan 2020 to 11 Jan 2023. As observed, both the curves of the United States (US) and the European Union (EU) had severe fluctuations, while those of China were relatively stable (Fig. 1, Fig. 2). Meanwhile, the number of cumulative confirmed COVID-19 cases and deaths in the US and the EU far exceeded China (Fig. 3, Fig. 4). It is noteworthy that this difference also exists between cities adopting different measures. As the most economically influential city in China, Shanghai faced a high risk of causing substantial losses to the local and national economy when imposing strict lockdown measures among the high-density and high-mobility population (Zhang et al., 2020); (Zhou et al., 2023). Given this situation, the local government initially adopted the policy of ‘‘precision prevention and control”: grid management was implemented without closing the city (Ma et al., 2022). However, statistics from the Shanghai Municipal Health Commission showed that the trend of the epidemic was beyond people’s imagination. Since 25 March 2022, the number of daily confirmed cases in Shanghai had exceeded 2000 and continued to increase exponentially. On the contrary, other mega-cities in China such as Qingdao and Shenzhen immediately took a series of more stringent emergency measures including prolonged blockade and mass testing, controlling their situation in a relatively short time (Fig. 5).

**Fig. 6:**
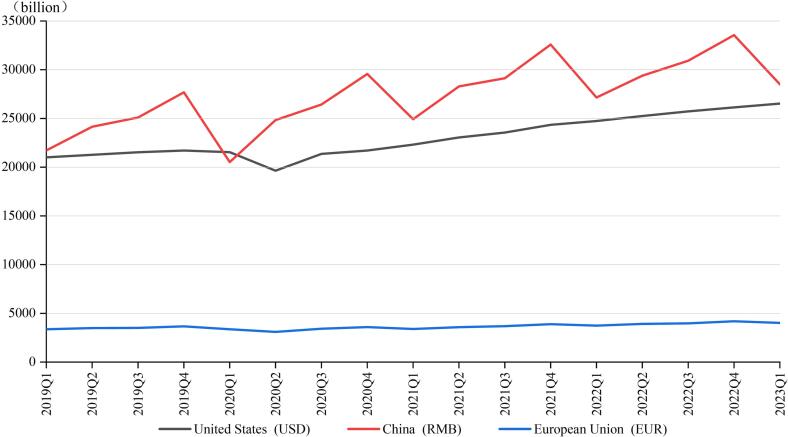
Quarterly Gross Domestic Product of United States, European Union and China, 2018–2023 **Source:** China National Bureau of Statistics, Eurostat and U.S. Bureau of Economic Analysis.

**Fig. 7:**
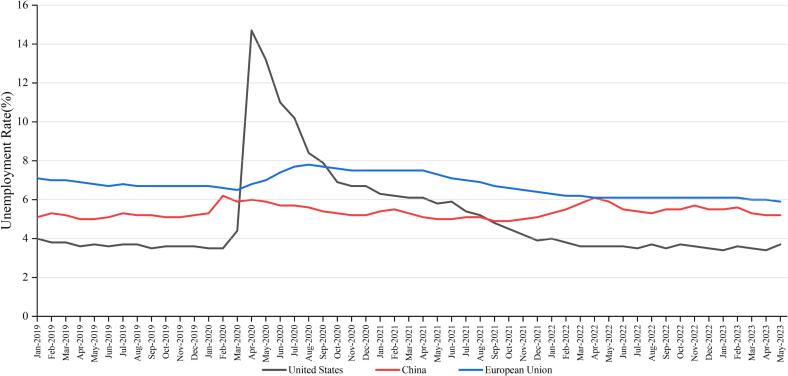
The monthly unemployment rate for United States, European Union and China, 2019–2023 **Source:** China National Bureau of Statistics, Eurostat and U.S. Bureau of Labor Statistics.

Although the dynamic zero-COVID policy can effectively prevent coronavirus spread with lower infection and mortality rates, its impact on the economy is nonetheless controversial. Some researchers proposed that implementing this policy would increase the economic and personal burdens (Bai et al., 2022); (Yeh et al., 2022). The uncertainty of timing and duration of lockdown might hamper asset investments and prevent companies from making long-term plans (Aghion et al., 2021); (Michie and Sheehan, 2022); (Zheng, 2023). Moreover, economists are concerned that draconian mobility restrictions not only disrupt China’s domestic economy but may also have ripple effects that touch every corner of the global economy (Liu, 2022); (Michie and Sheehan, 2022). On individual level, the dynamic zero-COVID policy may also not be friendly. Thousands of people were forced to take unpaid leave or even lose their jobs, meanwhile they still need to pay their rents and buy food to survive (Cheng, 2022); (Yeh et al., 2022). Although these measures might inevitably cause some economic losses in the short term, the resulting resumption of economic activity accumulates its benefit over time (Philippe and Marques, 2021); (Su et al., 2022). The advantage of continuity of economic activity could ultimately promote economic development from a long-term perspective. As shown in Fig. 6, the Gross Domestic Product of the US, the EU and China were growing at roughly the same pace. Notably, China contributed one-third of all imports and exports in the Asia-Pacific region and registered a record trade surplus in 2021 (Anukoonwattaka et al., 2022); (Li and Hong, 2022). Fig. 7 shows the unemployment rate in these countries and region from Jan 2019 to May 2023. As observed, the line representing the US reached a peak of 14.7 % in April 2020 while the data of China fluctuated slightly and was almost equal to that before the epidemic (Fig. 7).

## 3 Challenges to the dynamic zero-COVID policy

### 3.1 Superspreading potential and low pathogenicity of Omicron

The Omicron variant was first detected in South Africa on 24 November 2021 and then rapidly spread all over the world (World Health Organization., 2021). It is a highly divergent variant with significant sets of genetic mutations, which are associated with the superspreading potential, pathogenicity and re-infection risk (World Health Organization, 2022). Specifically, the substitutions on the receptor-binding motif (RBM) of Omicron (especially S477N, Q493R, G496S, Q498R and Y505H) result in the formation of new hydrogen bonds and salt bridges and more favorable surface properties, which make the spike acquire an increased RBM–angiotensin-converting enzyme 2 (ACE2) interaction network, leading to a strong affinity to ACE2 and enhanced transmissibility (Hong et al., 2022); (Laffeber et al., 2021); (Lan et al., 2020); (Mannar et al., 2021). T547K, N764K, and N856K in SD1 and S2 contribute to enhanced inter-protomer and S1–S2 interactions of Omicron. Thus, the predominantly populated (61 %) conformation for the Omicron S trimer is in a closed state with all the receptor-binding domain (RBD) epitopes buried, leading to conformational masking that prevents antibody binding and neutralization at sites of receptor binding (Hong et al., 2022). With that in mind, it is not surprising that Omicron variant is markedly resistant to neutralization by sera not only from individuals vaccinated with previous COVID-19 vaccines but also from convalescent patients infected with other variants (Ai et al., 2022); (Cele et al., 2022); (Dejnirattisai et al., 2022); (Edara et al., 2021); (Lu et al., 2022); (Zhang et al., 2022). Notably, although antibodies elicited by BA.1 infection after vaccination are enough to avoid severe infection with WT SARS-CoV-2 and BA.1, most of them are significantly evaded by BA.4 and BA.5 owing to F486V and L452R RBD mutations (Cao et al., 2022). What’s more, Omicron has faster and enhanced replication efficiency compared with the previous lineages. At 24 h after infection, the Omicron variant replicated over 70 times higher than both WT and Delta viruses in the human bronchi (Hui et al., 2022). The extent to which this rapid replication would contribute to transmissibility is unclear, but higher infectious virus load in conducting airways might lead to an increase in the amount of infectious virus released while speaking or breathing. The enhanced transmissibility of the Omicron variant significantly increased the number of infected individuals, which led the general public to think that complete elimination was no longer possible. Hence, the usefulness and sustainability of the dynamic zero-COVID policy has been questioned (Mo et al., 2023). Moreover, the attenuated replication of the Omicron variant in lung cells results in less severe clinical pneumonia compared with prior SARS-CoV-2 variants (McMahan et al., 2022). This is due to inefficient recognition by transmembrane serine protease 2 owing to its reduced efficiency of spike cleavage by host proteases (Shuai et al., 2022). Thus, among Omicron cases the percentage of asymptomatic infections is extremely high while the hospitalization and mortality rates are low (Jassat et al., 2022); (Wang et al., 2022). In the context of the huge drop of the harmfulness of the virus, people began to doubt the necessity of implementing the dynamic zero-COVID policy (Dai and Dai, 2022).

### 3.2 Psychological distress during the lockdown

In the past two decades, several studies have investigated the mental impact of quarantine due to epidemics, revealing that the experience of isolation is associated with psychological damage. For example, people quarantined after close contact with those who potentially have SARS reported 40.2 % for worry and 18.2 % for nervousness (Reynolds et al., 2008). Similarly, the prevalence of anxiety symptoms and feelings of anger was respectively 7.6 % and 16.6 % among individuals isolated during the Middle East Respiratory Syndrome (MERS) epidemic (Jeong et al., 2016). Moreover, even knowing someone quarantined for Ebola was associated with anxiety-depression and post-traumatic stress disorder (PTSD) symptoms (Jalloh et al., 2018). Like the past containment, the dynamic zero-COVID policy was also characterized by measures of social isolation and movement restrictions, leading to a negative impact on mental health (Lau et al., 2022); (Phiri et al., 2021). Moreover, the psychological impact of COVID-19 lockdown was highly heterogeneous across different social groups, contexts and countries (Prati and Mancini, 2021). Especially, it would be more serious for vulnerable people (e.g., children, adolescents, medical staff, the elderly) (Singh et al., 2021).

#### 3.2.1 Children and students

The degree of psychological distress during the COVID-19 pandemic of quarantined children was far greater than that of non-quarantined children. Worry, helplessness and fear were the most common feelings among them (68 %, 66 % and 61 %, respectively) (Saurabh and Ranjan, 2020). Moreover, there were differences in the severity of psychological symptoms of children who had experienced different isolation times. For example, compared to Spanish children, Italians felt sadder and lonelier after being isolated for a longer duration (Orgiles et al., 2020). Some parents chose to allow their children more time on electronic devices (Ayu Nuzulia et al., 2022). However, relieving negative emotions through this behavior may result in children’s addiction to electronic games (Niu et al., 2022). At the same time, the lockdown had led many universities to move to online teaching formats and cancel social activities and structured physical exercises (Rotnitsky et al., 2022); (Tria, 2020). Students were faced with major problems such as difficulty in communicating with lecturers, lack of access to laboratory and poor communication with peers, which significantly affected their learning experience (Selvanathan et al., 2020).

#### 3.2.2 Medical staff

As the care providers for the patients, medical staff were at high risk of developing serious mental health issues. In the absence of interpersonal communication and social support, they experienced burnout, lack of self-control and other mental health symptoms (Gupta and Sahoo, 2020). Moreover, in a study on MERS outbreak, Lee observed that the posttraumatic stress symptoms of quarantined healthcare workers could persist over time, especially sleep and numbness-related symptoms (Lee et al., 2018). Suicidal cases were also reported, as they faced accumulated psychological pressure and intense fear of falling sick or dying (Montemurro, 2020); (Rahman and Plummer, 2020). This is particularly worrying because physicians already had a higher suicide rate than the general population (Center et al., 2003).

#### 3.2.3 Elderly people

For many urban dwellers compulsorily quarantined at home, ordering daily items online became a safe alternative to shopping in-person (Huang, 2021). However, unlike younger generations, the elderly were not familiar with mobile computing technology such as smartphones and tablet computers and thus had difficulties in using them (Kim et al., 2022). In the meanwhile, difficulty meeting instrumental activities of daily living (such as home and financial management) in a virtual mode left them feeling like a “burden” to others, which led to a high degree of distress (Losada-Baltar et al., 2021). In China, 81.1 % of the residents aged 60 years and older suffered from at least one common chronic disease like asthma, diabetes and cardiopathy, and most are in need of constant medicine supply (Cheng, 2022); (Su et al., 2023). However, they could not get enough medicine due to the lockdown of hospitals and drug stores (Cheng, 2022). Moreover, strict isolation measures made it hard for patients to see a doctor (Li and Zhang, 2021). Many elderly people had to choose to reduce the amount of medication they take regularly and experienced widespread anxiety about discontinuation or termination of treatment (Brooks et al., 2020); (Cheng, 2022); (DurmuŞ and Durar, 2022).

## 4 Recommendations and suggestions after the end of lockdown

### 4.1 Mental health improvement

Although China has altered its policy in responding to the above challenges, there are still some problems to be solved in terms of mental health, vaccine development, drugs supply and economic recovery. The psychological effects of quarantine can last for months or even years. At four to six months after release from isolation, 6.4 % of 1,656 who had contact with the MERS patients but were not infected still felt angry while 3.0 % had anxiety symptoms (Jeong et al., 2016). Thus, it is necessary to place a high priority on those with a high risk for mental problems.

#### 4.1.1 Students

The provision of psychological intervention services could significantly improve and promote students’ mental health. After the pilot implementation of cognitive–behavioral interventions at a secondary school in Australia, the number of students in subclinical or clinical categories of depression decreased from 19.7 % to 6.9 % (Shochet et al., 2001). Thus, schools can train health counselors and offer classes within the school structure to improve both physical and mental health. Meanwhile, in order to help students to build a sense of spirit and relieve the pressure, they are encouraged to be active in the variety of campus activities (Shah et al., 2020). The social system should provide psychological services for teachers and parents, and establish a collaborative model of mental services which can gain a synergic effect in youth emotional support.

#### 4.1.2 Elderly people

With the relaxation of control measures, the number of infections has risen rapidly in mainland China (Huang et al., 2023). According to World Health Organization data, more than 95 % of COVID-19 deaths occur in people over 60 years old, which makes the elderly more prone to excessive spiritual and psychological anxiety (Fitriana and Kuncorowati, 2021). The government can establish a service team consisting of psychological health professionals, social workers and volunteers to provide mental health services for the elderly. At the same time, a series of lectures on mental health and group activities need to be organized to regularly evaluate the mental state of the elderly and implement interventions. Moreover, family members of elderly people living alone should be encouraged to keep in touch with them and provide adequate emotional support as much as they can.

#### 4.1.3 Medical health workers

Compared with non-medical health workers, medical health workers have shown higher prevalence rates of insomnia, anxiety and depression during the COVID-19 outbreak (Zhang et al., 2020). However, evidence-based assessments and mental health interventions for them are still relatively scarce (Lai et al., 2020). There is a need to provide targeted psychological support to avoid the occurrence of psychiatric illness in this population, such as timely counselling and screening and establishing a nationwide psychological support group. Moreover, health care workers are at a high risk of being stigmatized or distanced due to their vicinity to COVID-19 patients (Abdelhafiz et al., 2020). A more friendly social environment and mass media will help them gain the sense of self-efficacy, thus reducing stress and anxiety caused by stigmatization.

#### 4.1.4 Pregnant women

The prevalence of moderate to severe anxiety among pregnant women has arisen from 29 % before the pandemic to 72 % during its course (Davenport et al., 2020). More worryingly, another cross-sectional study of pregnant women reported that the risks of depression and self-harm thoughts increased by 20 % and 185 %, respectively, following the pandemic compared to before (Wu et al., 2020). It is well known that the anxiety level of women who participated in antenatal education was lower than that of women who did not (Paz-Pascual et al., 2008). Accordingly, healthcare professionals should pay timely attention to pregnancy and childbirth education, encouraging pregnant women to involve in social activities and planning physical exercise for them. Pregnant women can relieve their negative emotions by listening to music, watching movies, chatting with family members or friends and other ways to divert their attention.

### 4.2 Promotion of vaccination and research

COVID-19 vaccines successfully reduced the rates of infections and mortality (Mohammed et al., 2022). During the Omicron wave in Hong Kong, people who had not received a dose of vaccine accounted for 74 % of the deaths (Burki, 2022). The State Drug Administration should establish a rapid approval process for updated vaccines to maintain effectiveness against new COVID-19 variant. At the same time, the government should continuously deliver knowledge about safety and effectiveness of vaccines on social media platforms, so as to promote trust and increase vaccination coverage.

With the SARS-CoV-2 genome data been made available only a few weeks after the outbreak, bioinformatics platforms have become a key tool to gain time in the fight against the disease pandemic (Chukwudozie et al., 2021). Maintaining them is helpful to design potential vaccine candidates. Moreover, researchers developing vaccines should improve their ability to perform computational methods and use the high-throughput binding affinity prediction platforms formed by the combination of molecular dynamics, molecular docking and artificial intelligence to assess the effectiveness of vaccines.

### 4.3 Drugs supply and development

After lifting the lockdown, the demand for key drugs to mitigate COVID-19 symptoms has increased dramatically among the population (Kuo, 2023). Pharmaceutical enterprises and relevant departments need to expand their production of antipyretic analgesic and accelerate the research and development process of COVID-19 drugs. The government should balance the medical resources through transparent preparedness and contingency plans, active management and timely communications, which is crucial to address the drug shortage caused by medication misallocations. In the meantime it is also necessary to provide loans for the pharmaceutical enterprises and add drug development special fund.

### 4.4 Economic recovery and growth

Micro, Small, and Medium Enterprises (MSMEs) have been more affected by pandemic shock. For example, the financial restrictions for small-scale enterprises relative to large-scale enterprises increased by 0.188 units (Liao et al., 2023). The government can place state-owned enterprises as a buffer for MSMEs products and promote active cooperation between them to achieve a win–win situation. Moreover, financial incentives should be introduced to increase subsidies for damaged companies. Not only will digital technologies bring improvements in business model innovation, but it can also support supply chain business and economic resilience (Bai et al., 2021); (Klein and Todesco, 2021). Thus, the government could accelerate the digital transformation of MSMEs by training and investing in human resources, increasing labor-power competence, and strengthening data communication infrastructure (Bai et al., 2021); (Ordieres-Meré et al., 2020).

## 5 Conclusion

As discussed in our study, the dynamic zero-COVID strategy has obvious advantages in curbing the spread of SARS-CoV-2. The number of confirmed COVID-19 cases and deaths in countries and cities adopting this policy are far less than that in others trying to live with COVID-19. Moreover, although these measures might result in some economic losses in the short term, China still achieved a relatively healthy economic performance with sustainable economic growth and stable unemployment rate. However, the superspreading potential and low pathogenicity of Omicron brought a great challenge to the strict lockdown measures, making people question whether the dynamic zero COVID-19 policy should continue to be implemented. In addition, various psychological problems such as burnout, anxiety, depression and PTSD were more likely to appear in vulnerable groups which include children, students and the elderly. Although China has lifted the lockdown policy, there are still some issues that we have to overcome. The government should formulate and implement well-organized, coordinated, and structured psychology interventions to mitigate the negative impact of the previous lockdown on public mental health. Moreover, a sustained supply of therapeutic medications is supposed to be developed through active supply chain management, timely communications and transparent information. Given the continuous emergence of new variants, it is also desirable to promote the multichannel development of next generation vaccines and antiviral drugs. In addition, there is an urgent need to pay more attention to the MSMES that have been greatly affected. The government responsible for regulating economic development should actively steer collaborative cooperation between enterprises and provide support for the digital transformation to achieve sustainable economic growth.

## Disclosure of funding and conflicts of interest

The authors declare they have no competing interests. This work was supported by the Natural Science Foundation of Shandong Province (Grant No. ZR2020QC100).

## Declaration of Competing Interest

The authors declare that they have no known competing financial interests or personal relationships that could have appeared to influence the work reported in this paper.

## Data Availability

Data will be made available on request.
